# Exploring the Evolutionary Landscape with Targeted In Vivo Hypermutations

**DOI:** 10.3390/biomedicines14081831

**Published:** 2026-08-14

**Authors:** Thandava Vanapilli Nursimulu, Maryam Ali, Jumi A. Shin

**Affiliations:** Department of Chemistry, University of Toronto, 3359 Mississauga Road, Mississauga, ON L5L 1C6, Canada; t.vanapillinursimulu@mail.utoronto.ca (T.V.N.); maryaam.ali@mail.utoronto.ca (M.A.)

**Keywords:** directed evolution, continuous evolution, targeted mutagenesis, in vivo hypermutation, orthogonal replication, CRISPR-Cas, MutaT7, phage-assisted evolution

## Abstract

Directed evolution has revolutionized protein engineering by applying the principles of natural selection to the laboratory. However, traditional in vitro methods are quite labor-intensive, while common in vivo methods suffer from low mutation rates and high rates of off-target mutations. To address these issues, researchers have developed targeted mutagenesis tools for rapid in vivo evolution of biomolecules. In this review, we discuss recent in vivo hypermutation tools that enable rapid sampling of the vast evolutionary landscape, all while supporting simultaneous selection of the best proteins within living organisms. We focus on three main mechanisms of hypermutation: (i) orthogonal replication, which uses error-prone replication machinery to replicate the target gene with low fidelity; (ii) CRISPR-Cas-guided mutators, where mutagenic proteins are localized to virtually any user-defined loci; and (iii) transcription-coupled mutagenesis, a simple, yet elegant tool that exploits the innate processivity of orthogonal ribonucleic acid (RNA) polymerases to guide mutagenic proteins along the target gene during transcription. We highlight key advantages of these systems, as well as some clinically- and biotechnology-relevant applications. We discuss important limitations and how they could be addressed in the future to make hypermutation tools with broad mutational spectra and windows that span entire genes with minimal off-target effects.

## 1. Introduction

Over the course of a few billion years, nature has used evolution to create a diverse array of biomolecules with a vast range of functions. The evolutionary platform is indeed elegant and successful at producing molecules with desired function; it inspires us to harness evolutionary methods to create our own new molecules. One of the earliest demonstrations of laboratory evolution was the selection of rapidly replicating RNA molecules in vitro, culminating in the generation of the minimized replicating species termed Spiegelman’s Monster. This landmark experiment illustrated that biomolecules could evolve under controlled selective pressure outside of living systems [1]. Since then, directed evolution has enabled the development of proteins with enhanced catalytic activity, altered substrate specificity, improved stability and binding affinity through repeated cycles of mutation, screening or selection, and amplification.

Directed evolution has been traditionally performed using in vitro diversification techniques, such as error-prone PCR [2], DNA shuffling/recombination [3] and saturation mutagenesis (for more information, see refs. [4,5]). The resulting libraries from these techniques are subsequently screened to select for variants with desired traits, with enriched variants serving as templates for the next round of in vitro diversification, further advancing the evolutionary cycle.

**Figure 1:**
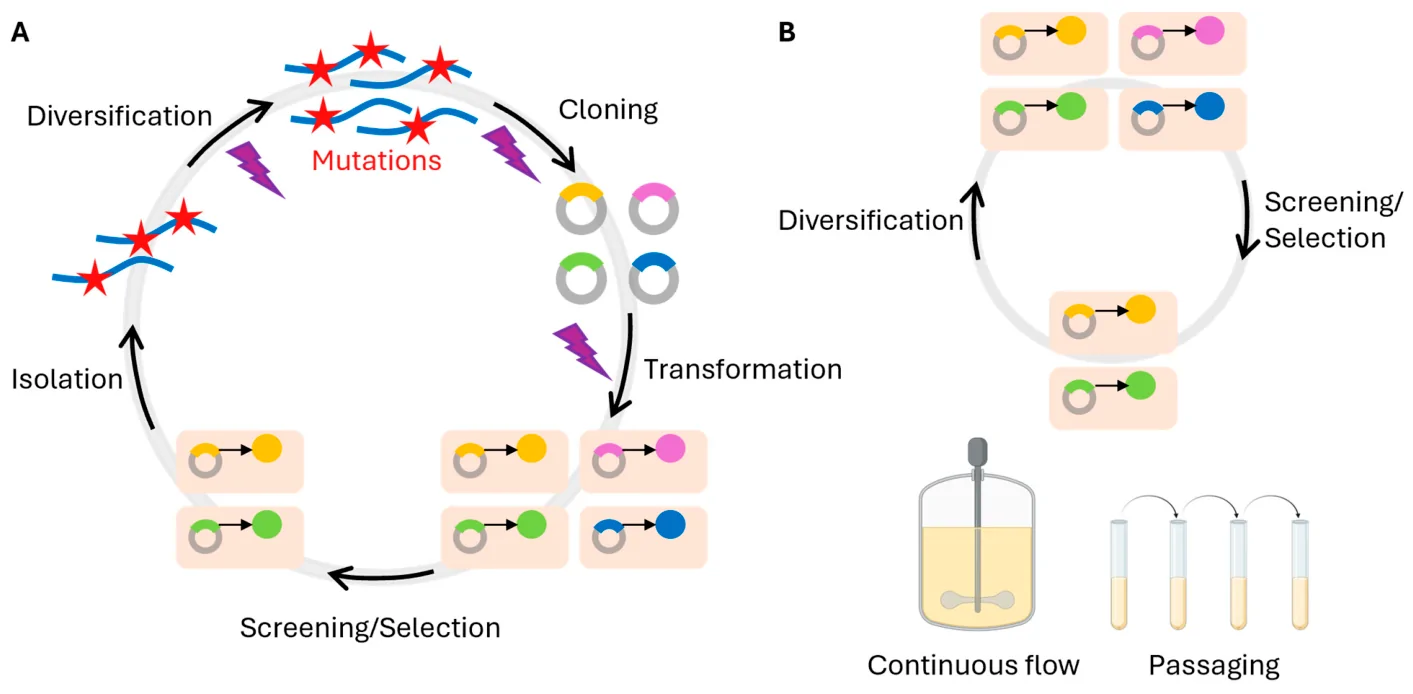
Comparison between in vitro and in vivo diversification methods. (**A**) Traditional directed evolution methods use in vitro diversification, which requires several labor-intensive steps (highlighted with purple lightning bolts), to generate variants that display different phenotypes (indicated by red stars and different colored genes and proteins), which can be screened/selected for desired activity. Multiple rounds of mutagenesis and selection are required to achieve marked increase in activity, so the target gene must be isolated and mutated again after selection. (**B**) To mitigate the labor- and time-intensiveness of in vitro methods, in vivo diversification platforms have been developed that allow simultaneous diversification and selection either in a continuous flow apparatus or by passaging cells under selective conditions.

Despite their success, traditional in vitro directed evolution workflows are often constrained by labor-intensive library construction, cloning, transformation, and screening steps (Figure 1A) [6]. Library size is frequently limited by transformation efficiency and screening throughput, restricting access to broader sequence space. In addition, iterative rounds of mutagenesis and re-cloning can slow evolutionary campaigns and increase experimental costs. Importantly, protein variants evolved exclusively in vitro may not fully capture the intracellular conditions required for optimal protein function, including native folding environments, post-translational modifications, and interactions with cellular machinery [7].

### 1.1. Harnessing Living Organisms for More Efficient Evolution

To address these limitations, in vivo evolution platforms were developed to generate diversity directly within living cells while also allowing concurrent functional selection (Figure 1B). Early approaches such as adaptive laboratory evolution (ALE) were designed to simulate natural selection in a controlled environment, enabling spontaneous accumulation of beneficial mutations that improved host phenotypes such as higher growth rates, stress tolerance, or improved metabolite production [8,9]. Additional strategies for in vivo global mutagenesis techniques employed mutator strains (XL1-Red) [10] and mutation plasmids [11], both of which have impaired DNA proofreading and repair mechanisms during DNA replication and thereby allow the accumulation of mutations over successive rounds of mutation cycles.

### 1.2. Restricting Mutagenesis to Target Genes Accelerates Evolution

While effective for generating mutations, global mutagenesis approaches often suffer from extensive off-target mutations, resulting in reduced genome stability, decreased cell viability, and the emergence of background mutations unrelated to the desired phenotype. In selection-based experiments, such unintended mutations can generate “cheater” phenotypes that can circumvent selective pressure without improving the gene-of-interest (GOI), thereby complicating downstream validation [7].

These challenges motivated the development of targeted in vivo mutagenesis platforms that localize mutagenic activity to a specific GOI, minimizing mutations elsewhere in the genome or vector backbone [12]. By concentrating mutations to the desired locus, such platforms improve library fidelity, preserve host fitness, and enable efficient exploration of sequence space in a physiologically relevant context [6]. Many of these systems are also compatible with continuous evolution workflows that allow diversification and selection to proceed simultaneously over multiple generations without repeated manual library construction (Figure 1B) [13].

Current targeted mutagenesis technologies generally employ one of several mechanistic strategies, including orthogonal DNA replication, CRISPR-guided recruitment of mutagenic enzymes, and transcription-coupled mutagenesis. Representative examples include OrthoRep, which uses an orthogonal error-prone plasmid replication system; EvolvR, which recruits an error-prone DNA polymerase to nCas9-targeted loci; and eMPAE, which combines phage-assisted evolution with transcription-coupled mutagenesis. In this review, we examine these systems as representative targeted in vivo mutagenesis platforms, and highlight their underlying mechanisms, strengths, limitations, and emerging applications in protein engineering. We further discuss key considerations in platform selection and future opportunities for continuous evolution.

## 2. Error-Prone Orthogonal Replication Accesses All Point Mutations Without Affecting the Host Genome

The first approach to targeted hypermutation involved an error-prone DNA Polymerase I (DNA Pol I) [14,15]. In contrast to the main replicative DNA Pol III of prokaryotes, DNA Pol I is only involved in the lagging-strand synthesis of chromosomal DNA and repair pathways. More importantly, DNA Pol I can be targeted to plasmids containing the ColE1 origin of replication. To increase the mutation rate, the fidelity of DNA Pol I was decreased by inactivating the 3′ → 5′ exonuclease activity [16,17]. Fabret et al. further improved the mutation rate of DNA Pol I by inactivating the mismatch repair pathway through deletion of *mutHLS* [14]. However, this approach may increase the genomic mutation rate due to the inactivation of a crucial DNA repair pathway; thus, in an effort to minimize such unwanted off-target effects, Camps et al. developed a similar system using a different error-prone DNA Pol I variant which is not only deficient in proofreading activity, but also favors misincorporation of nucleotides [15].

By inserting their GOI close to the ColE1 origin on the target plasmid, both groups reported comparable mutant frequencies of up to 1% of colonies screened with reporter assays. Although both approaches reported few genomic mutations, DNA Pol I is still involved in replication of part of the host genome and DNA repair; therefore, it is not orthogonal and could lead to the accumulation of genomic mutations over several generations in longer lasting evolution experiments.

**Figure 2:**
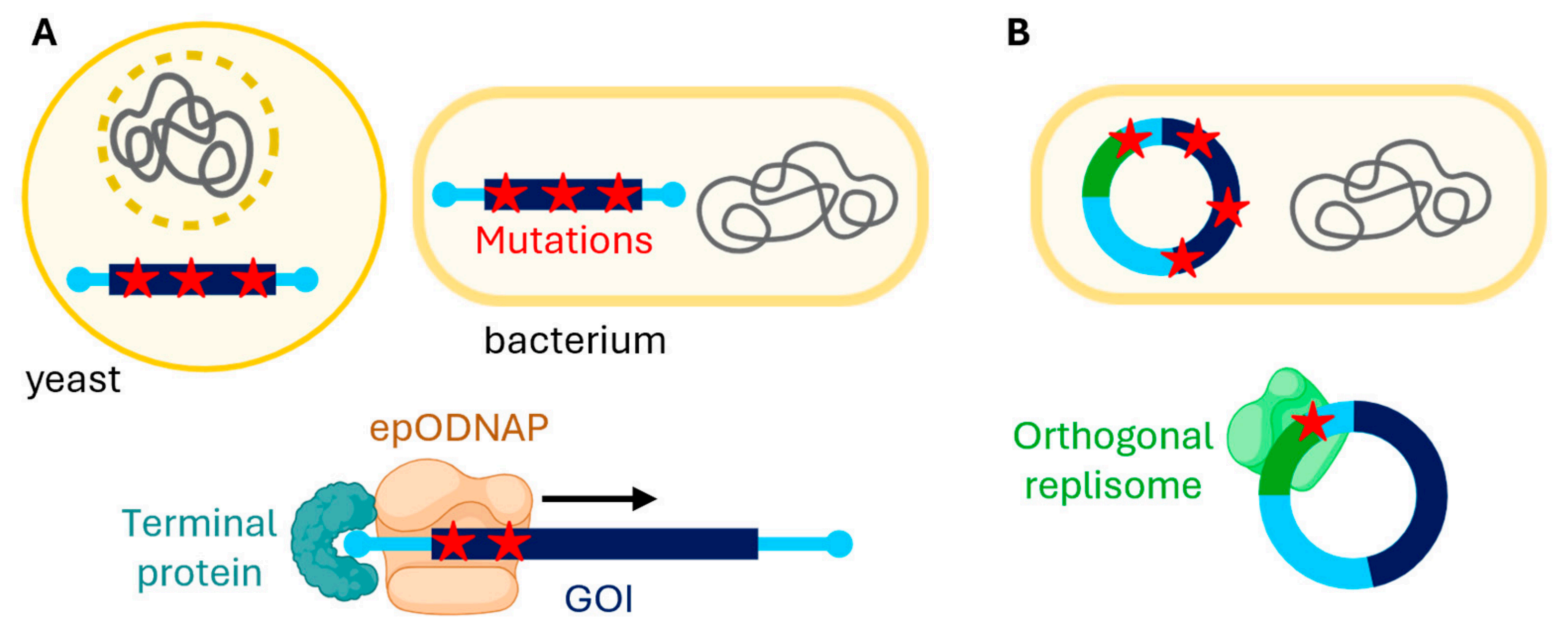
Orthogonal replication enables targeted mutagenesis. (**A**) Yeast-based OrthoRep and its bacterial variations use linear plasmid-terminal protein systems for their orthogonal mechanism of replication [18]. Terminal proteins (teal) prime replication and recruit the orthogonal error-prone DNA polymerase (epODNAP, tan), which randomly introduces point mutations (red stars) on the target plasmid. (**B**) The more common circular plasmids used in molecular biology can also benefit from orthogonal error-prone replication using orthogonal replisomes (green), such as with TADR [19] and T7 DNA polymerase in T7-ORACLE [20] and LySE [21].

To avoid unwanted genomic mutations, several orthogonal replication systems, consisting of linear plasmids or circular plasmids with orthogonal origins of replication and their associated DNA polymerases, have been developed. Such systems allow the rapid diversification of the GOI on the target plasmid while leaving the host genome unaffected (Figure 2).

### 2.1. Linear Plasmid Systems Allow Separation of Host and GOI Replication

In 2014, Chang Liu and colleagues reported OrthoRep in *Saccharomyces cerevisiae*, where they used the pGKL1/2 pair of linear, cytoplasmic plasmids of *Kluveromyces lactis*, which offers spatial separation from the host genome (Figure 2A) [18]. Replication of these linear plasmids is primed by terminal proteins instead of being initiated at an internal origin, a mechanistic difference from host DNA replication that further reinforces the orthogonality of this system. Moreover, since each plasmid is copied by its own replication machinery, they are mutually orthogonal, and thus, the GOI on each plasmid can be mutated independently [22]. After several rounds of engineering low fidelity variants of the pGKL1-specific DNA polymerase, the Liu group achieved mutation rates reaching 10^−4^ mutations/base pair (mut/bp), which is a million times greater than the genomic mutation rate [23,24]. More recently, OrthoRep has been adapted to allow automated continuous evolution [25] using the eVOLVER platform [26], and high-throughput selection of antibodies by combining yeast surface display and fluorescence-activated cell sorting [27,28].

Compared to the more common bacterial model organisms like *Escherichia coli*, *S. cerevisiae* grow more slowly and yield lower cell densities, thereby limiting the evolutionary speed and scale for similar culture volumes. Therefore, to benefit from the faster and denser growth of bacteria, orthogonal replication has been applied to *Bacillus thuringiensis* in BacORep [29], *E. coli* in EcORep [30,31], *Pseudomonas putida* and *Vibrio natriegens* in VinORep [32]. These variations in OrthoRep utilize the terminal protein-linear plasmid system of some bacteriophages and also achieve mutation rates exceeding 10^−4^ mut/bp. Although the lack of compartmentalization in bacteria prevents spatial separation of target plasmid from host DNA offered by the yeast OrthoRep, these systems still report negligible off-target mutations owing to the mechanistic difference in replication.

### 2.2. Orthogonal Origins of Replication and Replisomes

Linear plasmids, like the ones discussed above, tend to suffer from poorer transformation efficiencies relative to circular plasmids. To apply orthogonal replication to circular plasmids, Yi et al. and Diercks et al. developed methods that use a targeted artificial DNA replisome (TADR) [19] and the T7 replisome [20], respectively (Figure 2B). TADR is designed after the simple replisome of bacteriophage ΦX174, which uses only three components instead of the much larger and more complex replication machineries of other organisms. In TADR, ΦX174 CisA targeted nickase introduces a nick at an initiating sequence upstream of the GOI [19]. Then, *E. coli* Rep helicase binds to the nick site and unwinds DNA, while a low fidelity T5 DNA polymerase, fused to Rep, replicates the target plasmid and introduces random point mutations. In contrast, T7 orthogonal replisome-assisted continuous laboratory evolution (T7-ORACLE) uses the replication machinery of bacteriophage T7 to only replicate plasmids containing the T7 origin [20]. Diercks et al. engineered low fidelity T7 DNA polymerases by impairing proofreading and base selection. Both TADR and T7-ORACLE achieve a moderately high mutation rate of 10^−5^ mut/bp, though TADR also shows a 78-fold increase in off-target mutation rate.

Ong et al. also engineered low fidelity T7 DNA polymerase variants and fused nucleobase deaminases to increase the frequency of transition mutations [21]. They inserted a T7 packaging signal onto the target plasmid to allow both error-prone orthogonal replication and packaging of the plasmid into phages. Due to the lytic nature of T7 phages, the packaged phages can lyse and kill cells following transduction, which completely eliminates any potential off-target mutations. Their lytic selection and evolution (LySE) system also achieves a mutation rate of 10^−5^ mut/bp and boasts high control of mutagenesis and selection by tuning the amount of time for lysis and transduction.

To summarize, error-prone orthogonal replication uses low fidelity DNA polymerases that can introduce any point mutation during the replication of a specific plasmid without affecting the host genome. These polymerases also have a high processivity, which makes them useful for mutating long genes or entire gene clusters. However, this high processivity also means that error-prone orthogonal DNA polymerases cannot easily be restricted to a specific sequence on the target plasmid. Therefore, mutations can accumulate in selection markers or other undesirable genes on the target plasmid, which can create false positives and negatives.

## 3. CRISPR-Cas Is a Powerful Tool to Localize Mutations

**Figure 3:**
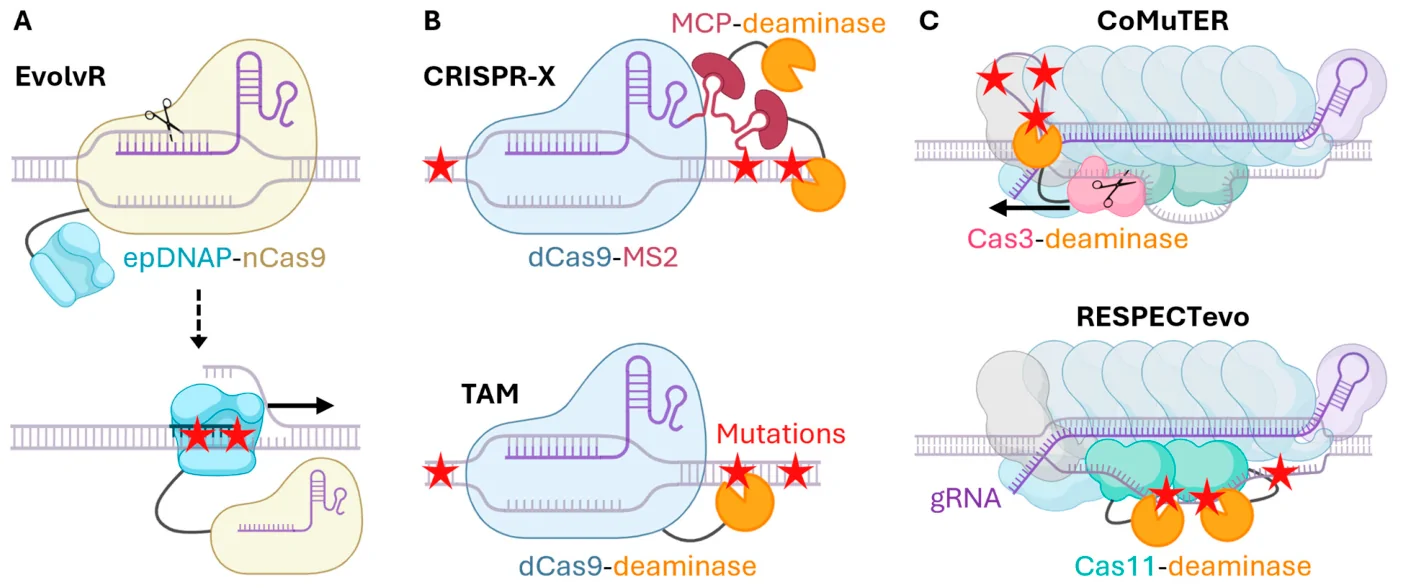
Overview of CRISPR-based mutagenesis tools. (**A**) EvolvR comprises nCas9 (olive), which introduces single-stranded breaks, and a fused error-prone DNA polymerase (epDNAP, cyan), which binds to the nick site and replicates the template strand with low fidelity [35]. (**B**) CRISPR-Cas can be used in combination with mutagenic enzymes to induce precise point mutations (red stars). CRISPR-X uses the MS2-MCP (red brown) system to recruit multiple base deaminases (orange) [36], while TAM uses dCas9-deaminase fusions (blue) to introduce more precise mutations [37]. (**C**) Multi-subunit Type I CRISPR systems can be used for targeted mutagenesis of regions spanning several kilobases with CoMuTER or specific regions of a protein with RESPECTevo. CoMuTER employs deaminases fused to the helicase-nuclease Cas3 (pink) [38], while RESPECTevo uses Cas11-deaminase fusions (teal) and varying gRNA (purple) lengths to tune the mutation window [39].

In the last two decades, the clustered regularly interspaced short palindromic repeats-CRISPR-associated protein (CRISPR-Cas) system, a prokaryotic defense mechanism against viruses, has garnered significant popularity in the field of genetic engineering due to its highly programmable nature and formidable versatility [33]. The CRISPR-Cas system comprises an RNA-guided endonuclease (e.g., Cas9 or Cas12) and a guide RNA (gRNA), which can be designed to bind to virtually any DNA sequence. Interestingly, various proteins can be fused to Cas to effect desired functions, such as transcription regulation [34] and even targeted mutagenesis (Figure 3) [33,35,36,37,38,39].

### 3.1. Targeted Error-Prone Replication with CRISPR-Cas

To localize error-prone replication to user-defined loci, Halperin et al. designed EvolvR by fusing error-prone DNA polymerases to nCas9, a Cas9 variant that nicks a single DNA strand [35]. When nCas9 binds to the DNA site complementary to its gRNA, nCas9 nicks the DNA and then dissociates (Figure 3A). The error-prone DNA polymerase binds to the nick site and replicates the intact strand with low fidelity and displaces the nicked strand, thereby introducing mutations downstream of the nick. The EvolvR system was further optimized by investigating various DNA Pol I mutants for enhanced mutagenesis activity and weakening the non-specific binding of nCas9 to DNA to facilitate dissociation of the fusion after nicking. To expand the mutation window, Halperin et al. improved the processivity of DNA Pol I by inserting the thioredoxin-binding domain of T7 DNA polymerase, and they achieve mutation rates up to 7.77 × 10^6^-fold above background or 10^−4^ mut/bp and mutation windows of ~60 bp in length in *E. coli* [35]. Alternatively, inherently more processive DNA polymerases, such as T5 and T7, can be implemented for significantly larger mutation windows [40].

Since then, EvolvR has also been applied in yeast [41,42], plants [43] and mammalian cells [44], which allows the evolution of proteins in their native environment. Since EvolvR is localized to desired loci by gRNAs, EvolvR can be used to evolve endogenous proteins and engineer organisms for specific functions, as seen in the development of EvolvR-based methods to engineer organisms for L-proline biosynthesis on an industrial scale [45,46].

Recently, Liu et al. proposed a variation of EvolvR that replaces Cas9 with the related, but more compact RNA-guided endonuclease OgeuIscB [47,48], Their OMEGA-R system also comprises a split endonuclease-DNA polymerase fusion that bioconjugates using the SpyCatcher/SpyTag system, which improves the expression levels of the mutator [49]. They achieved mutation rates up to 1.4 × 10^−5^ mut/bp/generation in both *E. coli* and *Bacillus subtilis* with extended mutation windows and less off-target mutations relative to EvolvR [47]. Both EvolvR and OMEGA-R are potent systems that can introduce all possible mutations at high frequencies and at very precise loci thanks to their gRNAs.

### 3.2. Introducing Precise Point Mutations Allows Specific Protein Regions to Be Mutated

In addition to DNA polymerase fusions, small mutation-inducing enzymes can also be fused to Cas to allow the introduction of mutations at specified loci (Figure 3B). Using this principle, base editors have been developed by fusing base deaminases and/or glycosylases to Cas proteins, primarily for therapeutic purposes and other gene editing applications [50]. However, these applications require base editors to be incredibly precise with very narrow editing windows of less than 5 bp, thereby limiting their potential as random mutagenesis tools in directed evolution.

Fortunately, Hess et al. and Ma et al. independently developed ways to work around this limitation with their CRISPR-X [36] and targeted AID-mediated mutagenesis (TAM) [37] systems, respectively. Both methods mimic somatic hypermutation, a process used by B cells to rapidly diversify antibodies [51]. CRISPR-X consists of a catalytically deficient Cas9 (dCas9), which only binds to DNA and possesses no endonuclease activity, and a gRNA harboring MS2 hairpins [36]. These hairpins recruit activation-induced cytidine deaminases (AID) via a fused MS2 coat protein (MCP) [52], which localizes the mutation-inducing enzyme and restricts its mutational activity close to dCas9 (Figure 3B). Cytidine deamination can result in (i) incorporation as C:G → T:A mutations, (ii) repair back to the original base, or (iii) triggering error-prone repair pathways as in somatic hypermutation, which allows any mutation at or near the deamination site. The MS2 system promotes the recruitment of multiple AID deaminases for every dCas9-gRNA binding to its target site, thus increasing the mutation rate and extending the mutation window up to 100 bp around the binding site. TAM uses the same principles as CRISPR-X but instead uses dCas9-AID fusions to achieve the same goal (Figure 3B) [37]. Overall, CRISPR-X and TAM enable in situ directed evolution in mammalian cells, bridging targeted genome editing and evolutionary diversity.

### 3.3. The Multi-Subunit Cascade Also Allows Targeted Mutagenesis

Class 1 CRISPR-Cas systems are significantly more abundant [53], yet less popular than class 2 systems, which include Cas9 and Cas12, despite several advantages of class 1 systems, such as their intrinsic gRNA processing capabilities and higher target site specificity [54]. In contrast to the single subunit class 2 endonucleases, all class 1 systems form large multi-subunit complexes, called CRISPR-associated complex for antiviral defense (Cascade) that process gRNAs and bind to DNA [55]. Some of the Cascade components, such as Cas3 which possesses both endonuclease and helicase activities, have been proposed to be promising tools for targeted mutagenesis (Figure 3C).

Zimmermann et al. developed confined mutagenesis using a Type I-E CRISPR-Cas system (CoMuTER) by fusing a cytidine deaminase to Cas3 to achieve targeted mutagenesis of an entire metabolic pathway comprising several genes in *S. cerevisiae* [38]. Following assembly of the Cascade complex, Cas3 unwinds and moves along the DNA. At the same time, Cas3 intermittently cuts the single-stranded DNA, while the fused cytidine deaminase randomly introduces point mutations (Figure 3C). CoMuTER allows targeted mutagenesis through a 55 kb region with an average 0.3 mutation/kb. A similar approach using the simpler Cas9 has also been developed in mammalian cells, where Cas9 is fused to both a dedicated helicase and a deaminase [56]. The high processivity of helicases is ideal for evolving entire metabolic pathways for biotechnology and synthetic biology purposes.

More recently, Lee and Kim reported their region-specific tool for continuous directed evolution (RESPECTevo), a system that utilizes Cas11 of the Cascade complex to localize deaminases more precisely compared to Cas3, while still maintaining a larger mutation window than CRISPR-X and TAM [39]. Cas11 plays a crucial role in stabilizing the Cascade complex, and as the length of the gRNA increases, more Cas11 units are required to bind to the complex [57]. Moreover, Cas11 is exposed to the single-stranded DNA in the DNA-gRNA hybrid, thereby providing easy access to fused deaminases (Figure 3C). RESPECTevo shows a high mutation rate of 1.5 × 10^−3^ mut/bp/day within a 200 bp region surrounding the Cascade complex, thereby allowing rapid mutagenesis of key regions of a protein, such as the active site of an enzyme.

Overall, CRISPR-Cas demonstrates its incredible versatility by serving as the source for the wide range of mutagenesis tools reviewed here. These tools employ a plethora of mutagenic enzymes to achieve efficient mutagenesis with mutation windows ranging from a few bases to several kilobases.

## 4. Transcription-Coupled Mutagenesis Allows Diversification of Large, Yet Specific, Regions

**Figure 4:**
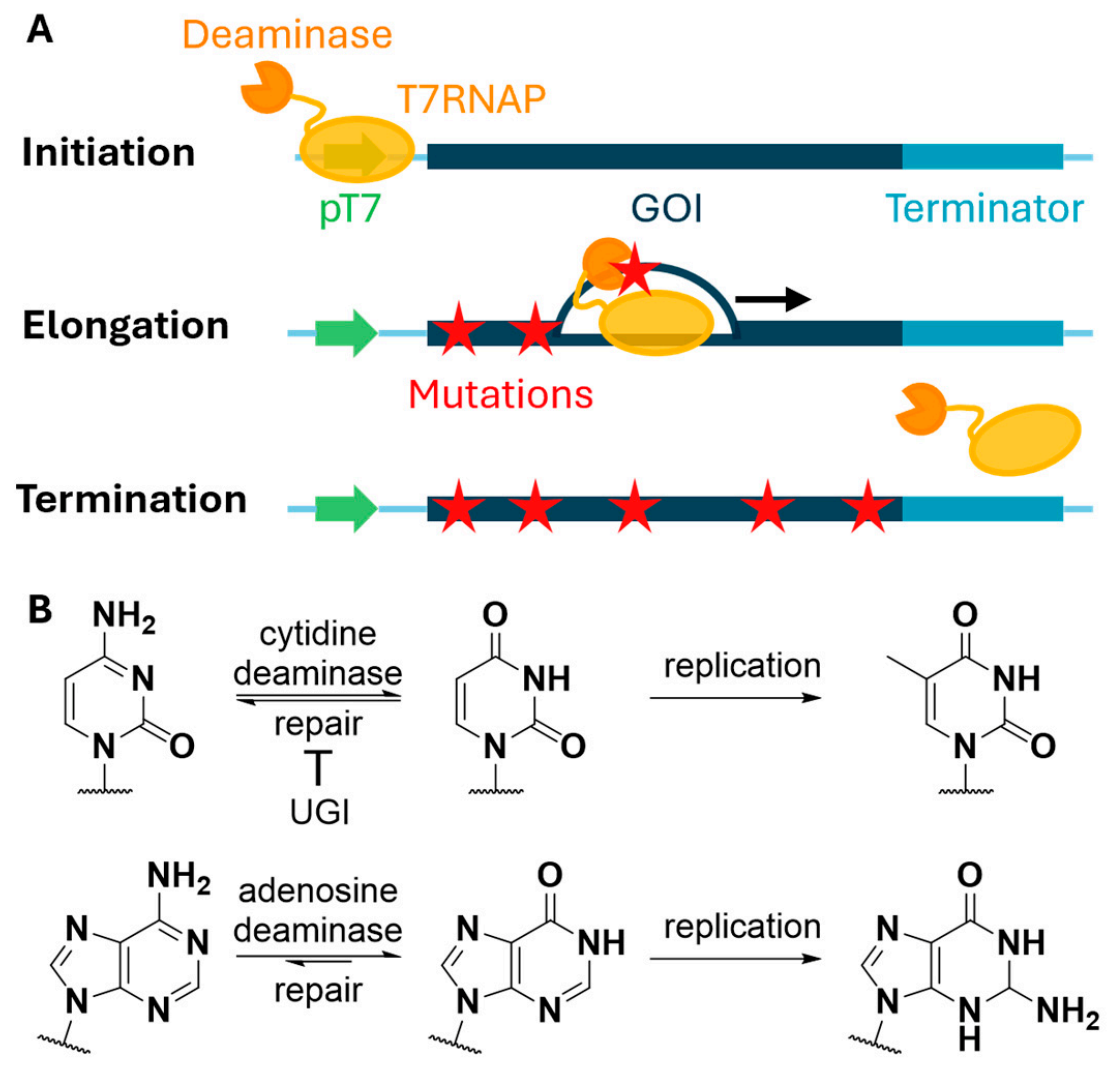
Overview of MutaT7. (**A**) The MutaT7 fusion comprising a base deaminase (orange) and T7RNAP (yellow) binds to the T7 promoter (pT7, green arrow) and initiates transcription of the GOI (dark blue box). The deaminase induces point mutations (red stars) in the exposed DNA in the transcription bubble. Upon reaching the terminator (teal box), the fusion dissociates from the target DNA, preventing downstream off-target mutations. (**B**) Base deaminases deaminate cytidine or adenosine to uridine or inosine, respectively. During replication, these noncanonical bases are misread by DNA polymerases as thymidine or guanosine, leading to C:G → T:A and A:T → G:C mutations. For efficient mutagenesis, the repair of uridine must be inhibited by UGI [58].

Taking inspiration from base editors, the Shoulders’ group pioneered the MutaT7 system, which consists of a base deaminase fused to the highly processive T7 RNA polymerase (T7RNAP) [58]. To allow localization of the base deaminase, the GOI was inserted between a T7 promoter and T7 terminator. The T7RNAP binds to its cognate T7 promoter and initiates transcription, which exposes single-stranded DNA for the base deaminase to mutate, and enables rapid and localized mutagenesis, as shown in Figure 4A. In the original MutaT7 system, the cytidine deaminase rAPOBEC1-T7RNAP fusion was integrated into the *E. coli* genome along with the deletion of *ung*, which encodes uracil *N*-glycosylase. Thus, deletion of *ung* prevents base excision repair of transient uridines upon cytidine deamination, thereby allowing C:G → T:A mutations to accumulate, since uridine is misread as thymidine during replication (Figure 4B). The Shoulders’ group demonstrated the effectiveness of their system using various antibiotic resistance assays. Since then, MutaT7 has been drastically improved with more efficient enzymes, such as *Pm*CDA1 [59,60], to achieve mutation rates up to 10^−4^ mut/bp and applied to human cells [61], yeast [62,63], plants [64], and other organisms [65,66,67,68].

The biggest breakthrough in the MutaT7 system, previously limited to cytidine deaminases, arrived in 2020 when Fernandez and colleagues introduced the first DNA-specific adenosine deaminase TadA* [69], which induces A:T → G:C mutations via inosine, a noncanonical base misread as guanosine during replication (Figure 4B) [59]. This new enzyme allowed broadening of the mutational spectrum, thereby allowing a deeper exploration of the evolutionary landscape. Since then, the more active TadA8e [70] has been incorporated into MutaT7 [68,71,72]. A yeast-optimized variant, yeTadA1.0, has also been engineered for TRIDENT (targeted in vivo diversification enabled by T7RNAP) [62].

To access all four transition mutations at approximately equal rates, two fusions, each of which contains a different deaminase, can be co-expressed as in eMutaT7^transition^ [72] or a single fusion containing both deaminases can be expressed [73]. More recently, TadDE, an enzyme capable of inducing all four transition mutations, has been added to the MutaT7 toolkit to create MutaT7^GDE^ [74]. Due to the simpler nature of this mutator, possible recombination between the two fusions of eMutaT7^transition^ and the slight bias observed in the double deaminase fusion can be avoided.

### 4.1. Recruitment of DNA Repair Proteins Accesses Transversion Mutations

Currently, base deaminases are the only mutation-inducing enzymes implemented in MutaT7, and they can only induce transition mutations, which represent a third of possible base substitutions and only ~20% of all possible amino acid substitutions, thereby restricting the evolutionary space accessible. However, in somatic hypermutation in B-cells, cytidine deamination triggers error-prone mismatch (MMR) and base excision repair (BER), leading to transversion mutations at the deamination site or at neighboring bases [75,76]. Cravens et al. mimicked this process by recruiting yeast DNA repair factors, namely MMR protein Msh6p [77] and BER protein Apn2p [78], to their TRIDENT fusion via protein–protein interactions between SH3 and SHL domains [62]. They achieved up to 20% non-C → T mutations, including transversion mutations such as T → G.

To further broaden the mutation spectrum and increase the efficiency of MutaT7, Huang et al. developed MutaT7^trans^ in yeast by directly fusing DNA repair proteins to T7RNAP, rather than recruiting these proteins via protein–protein interactions, which helped them achieve close to 40% non-C → T mutations [63]. Their MutaT7^trans^ constructs comprise *Pm*CDA-T7RNAP fused to one of three yeast DNA repair proteins: (i) MAG1, involved in BER [79], (ii) EXO1, involved in various pathways such as MMR and DNA double-strand break repair [80], and (iii) REV3, involved in translesion synthesis among other pathways [81]. These repair proteins both enhance the mutation rate by up to two-fold and allow accessing transversion mutations. However, only *Pm*CDA1 has been implemented into MutaT7^trans^, which restricts transition and transversion mutations at C or G bases only. Therefore, implementing adenosine or general deaminases will significantly improve MutaT7^trans^ by accessing all twelve possible point substitutions.

### 4.2. Stopping T7RNAP Is Critical to Preventing Downstream Mutations

Since T7RNAP is highly processive, it is imperative that the fusion dissociates at the terminator to prevent unwanted off-target downstream mutations. The Shoulders’ group discovered that a simple array of four consecutive native T7 terminators was sufficient to effectively stop the deaminase-T7RNAP fusion [58]. Alternatively, our group has employed the shorter, but highly efficient, synthetic T7 terminator, T7hyb10 [82,83]. However, if the GOI is part of a larger construct that must be expressed or terminators are undesirable, dCas9 roadblocks can be used [59]. Up to three of these dCas9 roadblocks may be needed on both DNA strands to effectively halt T7RNAP.

### 4.3. Enhancing the Applicability of MutaT7

Eukaryotes, especially those that are multicellular, have typically suffered from a lack of efficient hypermutation tools, which has hampered the directed evolution of eukaryotic proteins since they have mostly been evolved in non-native environments [84]. Utilizing the highly efficient and orthogonal MutaT7 platform, the Chen group developed T7 polymerase-driven continuous editing (TRACE) to allow continuous targeted mutagenesis in human cells [61]. They fused T7RNAP to both cytidine deaminase AID*Δ and uracil *N*-glycosylase inhibitor (UGI), which inhibits UNG to prevent the repair of uridine to cytidine. Chen et al. used TRACE to study the emergence of drug resistance in mitogen-activated protein kinase kinase 1 (MEK1/MAP2K1), a protein involved in many cellular processes, such as transcription regulation [85]. Continuous TRACE mutagenesis of MEK1 under drug selection enabled the enrichment of drug-resistant variants, including both clinically relevant mutants as well as novel mutants. Similarly, Cravens et al. applied the MutaT7 system to yeast to create TRIDENT [62], while Butt et al. established MutaT7 in stable transgenic plants to study herbicide resistance [64]. Continuous targeted mutagenesis in plants unlocks the potential to engineer plants to become more resistant to climate change and pests, as well as increasing crop yields.

The use of MutaT7 in several other organisms, both model [66,68,86] and non-model [65,67], has also been explored to facilitate the engineering of various prokaryotes for biotechnology purposes. For example, Velázquez et al. tested MutaT7 in *P. putida*, a bacterium found in soil that can survive under strong redox and other harsh conditions, which can be promising qualities for biotechnology applications [65]. However, they observed a much lower mutation rate in *P. putida* (only 566-fold above control) compared to well over 1000-fold higher rates in *E. coli*, likely due to the lower effectiveness of T7RNAP in other hosts.

To make MutaT7 more efficient in various hosts, Shao et al. replaced T7RNAP with other orthogonal phage RNAPs, namely MmP1, K1F and VP4, which show much higher transcription efficiencies in various host organisms [67]. They tested their orthogonal transcription mutators (OTM) in both *E. coli* and non-model *Halomonas bluephagenesis*, a halophile capable of producing various types of polymers and biomolecules under non-sterile conditions [87], and achieved mutation rates 10^6^-fold above background or 10^−3^ mut/bp/day. These orthogonal RNAPs are highly specific for their own promoters, meaning that they show minimal crosstalk with other promoters. This feature can be exploited by fusing different deaminases to different RNAPs for more balanced mutations and to avoid recombination from eMutaT7^transition^ or biases from fusions containing two deaminases.

### 4.4. Growth-Coupled MutaT7-Mediated Directed Evolution

Most MutaT7 systems have been tested on simple antibiotic resistance genes, where expression of these genes confers resistance to the antibiotics added to the media, thereby allowing cells to grow. However, for proteins with other functions, greater care must be taken when designing selection strategies to link protein activity to growth, while maintaining the advantageous high throughput nature of in vivo directed evolution and avoiding laborious screening assays. For example, Ting et al. linked the activity of *Mesorhizobium loti* carbonic anhydrase (MlCA) to *E. coli* growth using CRISPR-interference and direct enzymatic performance evaluation and determination (DEPEND) [88,89]. After generating random mutations using MutaT7, the authors selected the highest performing MlCA variants in a high throughput manner using DEPEND.

Another approach is growth-coupled continuous directed evolution (GCCDE), developed by Deng et al., where MutaT7 induces mutagenesis while enzyme activity is linked to growth in selective medium in a bioreactor [90]. By coupling enzyme catalytic performance to host fitness, the system enables real-time selection of beneficial variants without iterative manual screening. The authors validated GCCDE by evolving the thermostable CelB enzyme from *Pyrococcus furiosus* for enhanced β-galactosidase activity at lower temperatures. Variants that metabolized lactose promoted faster bacterial growth and were enriched in a continuous culture containing over 10^9^ evolving cells. Overall, these two strategies streamline enzyme engineering by bypassing traditional cycles of in vitro mutagenesis, cloning, and screening, and provide a broadly applicable framework for optimizing diverse enzymes through growth-based selection.

### 4.5. Host Replacement Eliminates Genomic Mutations

**Figure 5:**
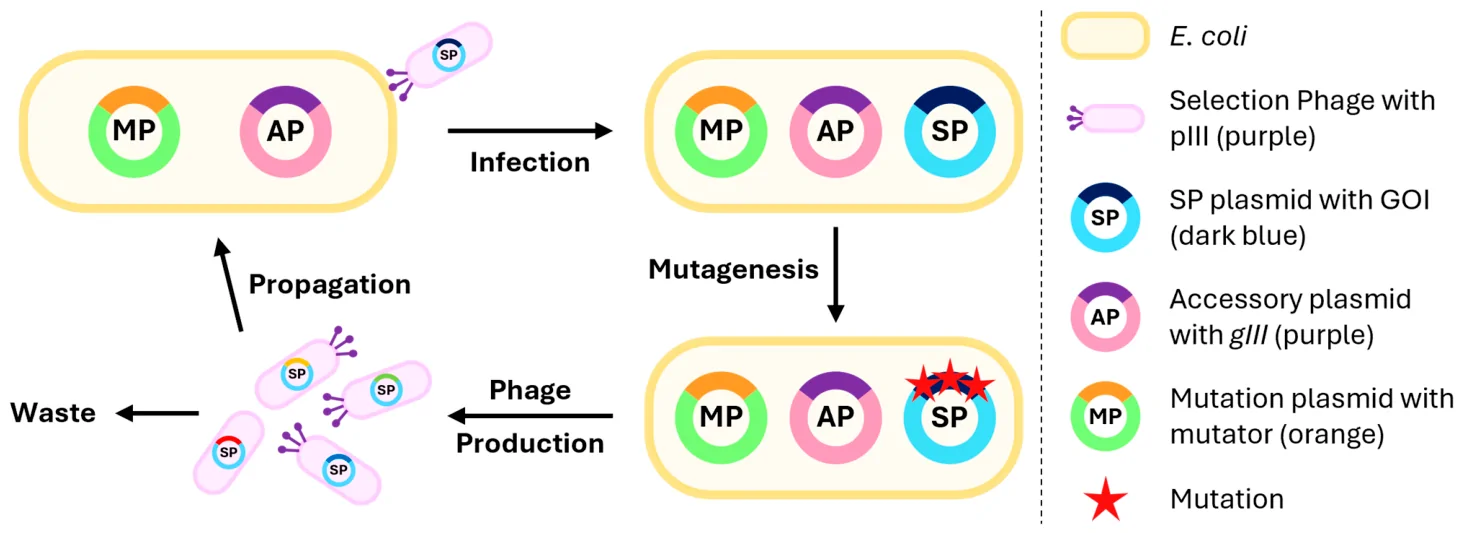
Phage-assisted evolution exploits the short lifecycle of M13 phages for rapid evolution. Phage-assisted evolution is either performed in a continuous flow apparatus or by serial dilution of selection phages [92]. In both cases, fresh host cells are added to phages, which infect cells. Once the phage DNA (SP plasmid) is injected into the cell, it replicates and undergoes simultaneous mutagenesis due to mutagenic proteins encoded on the mutation plasmid. In eMPAE, mutations are limited to the GOI on the SP plasmid, creating variants whose activity is linked to the expression of the essential *gIII* from the accessory plasmid [83]. Only active mutants produce infectious phages that can infect new cells, while less active mutants produce less infectious phages and are discarded.

Our group designed enhanced mutation phage-assisted evolution (eMPAE) by combining phage-assisted evolution with MutaT7, which, to our knowledge, is one of the first virus-based directed evolution methods capable of targeted mutagenesis [83]. Phage-assisted evolution was developed by David Liu and coworkers by looping the four steps of directed evolution into the M13 bacteriophage life cycle [91,92]. In this system, the GOI is inserted into the selection phage (SP), which is a modified M13 bacteriophage lacking *gIII* that encodes a phage coat protein (pIII) necessary for phage propagation (Figure 5). This essential phage gene is instead provided on an accessory plasmid (AP) and *gIII* expression is linked to the activity of the protein-of-interest. Host cells also carry a mutation plasmid (MP) that encodes proteins that induce mutations in the GOI. Mutants with greater activity promote greater *gIII* expression, which in turn leads to more phage propagation, whereas mutants with lower activity are capable of little-to-no phage propagation and are washed out through serial dilutions or in a continuous flow apparatus (Figure 1B and Figure 5).

Phage-assisted evolution typically uses the mutation plasmid MP6, which mutates all DNA in host cells. In our initial attempt at phage-assisted evolution on ME47, a small protein drug designed to inhibit the proto-oncogene Myc, we observed a low on-target mutation rate with MP6 [93,94]. To specifically target more mutations to the GOI, our group integrated MutaT7 into phage-assisted evolution by flanking the GOI with a T7 promoter and the T7hyb10 terminator on the SP plasmid [83]. Using our mutation plasmid MPT7 and modified SP plasmid, we achieve high gene specificity with mutation rate 5.6 mutations/kb/day. Our system also reports the off-target rate < 6.2 × 10^−3^ mutations/kb/day; this low rate of mutations outside of the GOI can be ascribed to the 98% efficient T7hyb10 terminator that plays a critical role in preventing read-through transcription, ultimately limiting the risk of creation and propagation of cheater phages that can circumvent selective pressure [82]. We also determined that the region between the T7 promoter and T7 terminator should be >800 bp to prevent premature dissociation of T7RNAP when it encounters the highly efficient terminator. Thus, smaller GOIs should include a spacer region before the terminator for efficient mutagenesis.

A key advantage of our system relative to other MutaT7 systems is that host cells in eMPAE are constantly discarded and replaced by fresh cells, meaning that any off-target genomic mutations are eliminated. This is critical for long evolution campaigns, where cheaters can poison the experiment, and/or when using hyperactive deaminases, such as TadA8e, which have an elevated rate of off-target mutations [71,72]. Furthermore, multiple mutators can be employed in different host cells to accumulate diverse mutations: this conveniently circumvents the need to co-transform multiple mutation plasmids into a single host cell. Since phages allow horizontal gene transfer, mutations introduced by each deaminase in different cells can accumulate sequentially within the same SP plasmid carrying the GOI. This feature of eMPAE can reduce the stress on the bacterial host compared to most methods that do not use phages, avoiding trade-off between achieving high mutation rate and maintaining mutational diversity.

By combining the MutaT7 tool and the horizontal gene transfer capabilities of phages, eMPAE provides a highly efficient platform for rapid gene diversification and directed evolution of proteins with minimal user intervention.

## 5. Conclusions/Outlook

The development of targeted in vivo hypermutation platforms has transformed directed evolution by enabling continuous diversification of genes within living cells, while minimizing unwanted mutations elsewhere in the genome. Unlike traditional in vitro approaches, these in vivo evolution systems seamlessly integrate mutagenesis, selection, and replication in a continuous workflow, reducing experimental complexity and allowing proteins to evolve within their native cellular context. The strategies discussed in this review demonstrate the diverse approaches developed to localize mutagenesis in a broad range of hosts, with platforms such as orthogonal DNA replication, CRISPR-guided recruitment of mutators, and transcription-coupled mutagenesis.

Each strategy offers distinct advantages and trade-offs, as summarized in Table 1. Orthogonal replication systems such as OrthoRep provide high mutation rates and the broadest mutational diversity by accessing all point mutations, though they cannot be restricted to the target gene(s) on the plasmid [19,24]. CRISPR-based systems offer higher precision, but their mutation windows and/or mutational spectra are often constrained by the properties of the mutators employed [35,36,37,39]. Transcription-coupled platforms such as MutaT7 achieve a favorable balance between large mutation windows and high mutational efficiency [58,72,74]. However, these transcription-coupled mutagenesis strategies are mostly limited to transition mutations [63]. The recent integration of targeted mutagenesis systems into phage-based evolution methods, as in eMPAE and LySE, demonstrates how the emergence of occasional off-target genomic mutations can be avoided by packaging the evolving target biomolecule into phages, followed by transduction into fresh cells [21,83].

Despite remarkable progress, several challenges remain: no current platform achieves complete mutational diversity, mutation windows limited to one or more GOIs, and virtually no off-target activity. Future advances in this field should focus on expanding accessible mutational space by using a tool capable of introducing all point mutations, improving target specificity, and implementing robust selection strategies to efficiently select for promising protein variants: such improvements will further enhance efforts to evolve proteins with enhanced structure and function. For example, the MutaT7/OTM platforms will benefit from more active DNA repair proteins or glycosylases to access transversions more efficiently. While the small mutation windows of CRISPR-based methods, like EvolvR and RESPECTevo, can be useful for focusing mutagenic power to key regions of a protein, they would greatly benefit from mutation windows that span an entire GOI, as the key advantage of directed evolution over rational and computation-based protein design is that directed evolution can uncover completely unexpected mutations.

## Figures and Tables

**Table 1:** Summary of in vivo targeted hypermutation methods.

Technique	Mutagenesis Mechanism	Mutation Spectrum	Mutation Window	Mutation Rate *	Host Organism	References
OrthoRep	Error-prone orthogonal DNA polymerase and linear plasmid	All point mutations	Entire target orthogonal plasmid	10^−4^ mut/bp	*S. cerevisiae*, *E. coli*, other prokaryotes	[18,24,31,32]
TADR & T7-ORACLE	Error-prone orthogonal DNA replisome and circular plasmid	All point mutations	Entire target orthogonal plasmid	10^−5^ mut/bp	*E. coli*	[19,20]
LySE	Error-prone T7 replisome coupled with base deaminases and T7 phage packaging	All point mutations (higher frequency of transitions)	Entire target orthogonal plasmid	10^−5^ mut/bp	*E. coli*, T7 phage	[21]
EvolvR	nCas9-guided error-prone DNA polymerase	All point mutations	~60 bp downstream from nick site	10^−4^ mut/bp	*E. coli*, yeast, plants, mammalian cells	[35]
OMEGA-R	OgeuIscB-guided error-prone DNA polymerase	All point mutations	Hundreds of bp	10^−5^ mut/bp/gen	*E. coli*, *B. subtilis*	[47]
CRISPR-X and TAM	dCas9-deaminase	Transitions, some transversions	Up to 100 bp around binding site	10^−3^ mut/bp	Mammalian cells	[36,37]
CoMuTER	Cas3-deaminase fusion	Transitions only	Up to ~55 kb	10^−4^ mut/bp	*S. cerevisiae*	[38]
RESPECTevo	Cas11-deaminase fusion	Transitions only	~200 bp	10^−3^ mut/bp/day	*E. coli*	[39]
MutaT7	Base deaminase fused to T7 RNA polymerase	Transitions only	Up to 10 kb between T7 promoter and T7 terminator	Up to 10^−4^ mut/bp	*E. coli*, *S. cerevisiae*, plants, mammalian cells, other prokaryotes	[58,59,60,61,62,64,65,66,68]
MutaT7^trans^	MutaT7 coupled with DNA repair proteins	Transitions; some transversions	~2 kb between T7 promoter and T7 terminator	10^−3^ mut/bp	*S. cerevisiae*	[63]
OTM	Base deaminases fused to phage RNAPs	Transitions only	~2 kb between promoter and terminator	10^−3^ mut/bp/day	*E. coli*, *H. bluephagenesis*	[67]
eMPAE	MutaT7 coupled with phage-assisted evolution	Transitions only	~1 kb between T7 promoter and T7 terminator	10^−3^ mut/bp/day	*E. coli*, M13 phage	[83]

* Mutation rates have been measured using different methods, leading to different units.

## Data Availability

No new data were created or analyzed in this study. Data sharing is not applicable to this article.

## Abbreviations

The following abbreviations are used in this manuscript:

RNA	Ribonucleic acid
PCR	Polymerase chain reaction
DNA	Deoxyribonucleic acid
ALE	Adaptive laboratory evolution
GOI	Gene-of-interest
CRISPR	Clustered regularly interspaced short palindromic repeats
Cas	CRISPR-associated protein
eMPAE	Enhanced mutation phage-assisted evolution
epODNAP	Error-prone orthogonal DNA polymerase
bp	Base pair
TADR	Targeted artificial DNA replisome
T7-ORACLE	T7 orthogonal replisome-assisted continuous laboratory evolution
LySE	Lytic selection and evolution
gRNA	Guide RNA
epDNAP	Error-prone DNA polymerase
MCP	MS2 coat protein
TAM	Targeted AID-mediated mutagenesis
CoMuTER	Confined Mutagenesis using a Type I-E CRISPR-Cas system
RESPECTevo	Region specific tool for continuous directed evolution
Cascade	CRISPR-associated complex for antiviral defense
kb	Kilobase pair
RNAP	RNA polymerase
pT7	T7 promoter
UNG	Uracil *N*-glycosylase
UGI	Uracil *N*-glycosylase inhibitor
TRIDENT	Targeted in vivo diversification enabled by T7RNAP
MMR	Mismatch repair
BER	Base excision repair
TRACE	T7 polymerase-driven continuous editing
OTM	Orthogonal transcription mutator
MlCA	*Mesorhizobium loti* carbonic anhydrase
DEPEND	Direct enzymatic performance evaluation and determination
GCCDE	Growth-coupled continuous directed evolution
*gIII*	M13 *gene III*
SP	Selection phage
AP	Accessory plasmid
MP	Mutation plasmid
